# Unilateral temporary diaphragmatic paralysis secondary to bronchial artery embolization in a girl with cystic fibrosis and massive hemoptysis: a case report

**DOI:** 10.1186/s12890-020-1076-3

**Published:** 2020-02-11

**Authors:** V. Terlizzi, M. Botti, G. Gabbani, F. Fanelli, M. De Martino, G. Taccetti

**Affiliations:** 1 Cystic Fibrosis Centre, Department of Pediatric Medicine, Anna Meyer Children’s University Hospital, Viale Gaetano Pieraccini 24, 50139 Florence, Italy; 2 Department of Pediatrics, Santa Chiara Hospital, University of Pisa, Pisa, Italy; 3 Department of Emergency, Diagnostic and Operative Radiology, Careggi University Hospital, Florence, Italy; 4 Department of Health Sciences and Anna Meyer Children’s University Hospital, University of Florence, Florence, Italy

**Keywords:** Children, Cystic fibrosis, Phrenic nerve, Massive hemoptysis

## Abstract

**Background:**

Massive hemoptysis is a serious complication in Cystic Fibrosis (CF), occurring commonly in older patients. Bronchial artery embolization (BAE) can be performed to stop the bleeding. BAE is generally safe and effective, but can sometimes lead to serious complications. We report the first case of temporary unilateral diaphragmatic paralysis associated to lung consolidation following BAE in a pediatric CF female patient. This complication worsened the lung function of the patient who underwent lung transplantation after 9 months.

**Case presentation:**

A 14 years old female CF patient followed by the CF center of Florence presented low-grade fever, cough increase and recurrent episodes of major hemorrhages such as to carry out a BAE. Within 24 h the patient started to complain of severe thoracic pain in the right hemithorax, increased dyspnea and fever. A computed tomographic angiography and a dynamic fluoroscopic evaluation revealed the right diaphragmatic paralysis, not present before the procedure. After 4 days the clinical condition and radiological imaging had improved with restored mobility of the right hemidiaphragm. Nine months later, she required mechanical ventilation, and subsequently the initiation of extracorporeal membrane oxygenation (ECMO) for a pulmonary exacerbation with septic shock. Lung transplantation in ECMO was performed with success.

**Conclusion:**

Clinicians should be aware of the possibility of phrenic nerve injury with BAE in pediatric CF patients.

## Background

Hemoptysis is a common complication in Cystic Fibrosis (CF) occurring in approximately 9% of patients.^1^ The definition currently used for massive or major hemoptysis is acute bleeding of more than 240 ml/day or recurrent bleeding of substantial volumes over several days.^1, 2^ In the pediatric CF population it occurs in approximately 1 to 1.5% of cases and is a potentially life-threatening event.^1^ Percutaneous bronchial artery embolization (BAE) is a safe and effective treatment in patients with major hemoptysis.^3^ The CF Foundation guidelines support BAE for life-threatening hemoptysis without age limits.^2^ Chest pain and dysphagia are commonly reported after BAE. Serious complications are rare; one of these being non-target vessel embolization with subsequent tissue ischemia.^3^ We present a pediatric case of massive hemoptysis treated with BAE and complicated by unilateral diaphragmatic paralysis.

## Case presentation

**Fig. 1:**
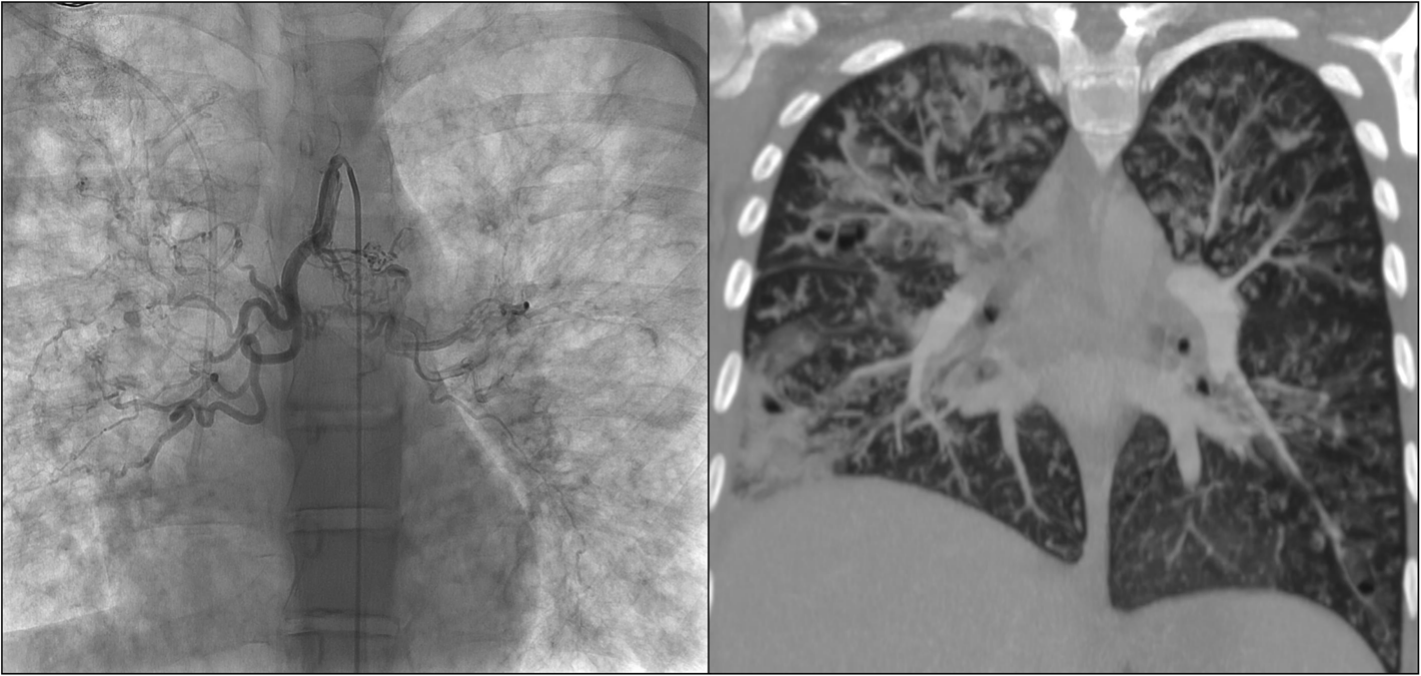
**a)** angiography of the main common bronchial artery, **b**) MIP coronal MDCT with elevated right hemidiaphragm and concomitant lung consolidation

A female CF patient followed by the CF center of Florence had the first episode of hemoptysis at the age of 14 years. In the previous 2 years she suffered from chronic respiratory failure with a Forced Expiratory Volume in 1 s (FEV_1_) of 40–45% and chronic colonization by methicillin-resistant *Staphylococcus aureus* and intermittent colonization by *Pseudomonas aeruginosa*. At home, she presented low-grade fever, cough increase and two episodes of hemoptysis (< 100 ml/day) treated initially with rest, minocycline and rifampicin administered orally. In the subsequent hours persisted others episodes of hemoptysis, and consequently she was referred to the Pediatric Emergency Department. On admission she was clinically stable. Intravenous antibiotic (linezolid and ceftazidime) were started. During the following 2 days she continued to have major hemorrhages (> 200 ml/day). Computed tomographic angiography (CTA) of the chest did not show any active bleeding, although bronchial arteries were very tortuous and ectasic. The high risk of rebleeding, due to the severe vascular lung condition, led us to perform a percutaneous embolization. Selective bronchial angiography revealed the presence of a shunt between the right and left bronchial arteries. A super-selective catheterization of the right bronchial artery was achieved using a 2.4F microcatheter (Fig. 1a). Embolization was conducted using spherical microparticles (700–900 μm) until a “stop-flow” was evident in the distal segment of the artery.

No further episodes of hemoptysis were observed after BAE. However, within 24 h the patient started to complain of severe thoracic pain in the right hemithorax, increased dyspnea and fever. For these reasons, a CTA was repeated showing an elevated right hemidiaphragm, an area of consolidation in the lateral segment of the right lower lobe and a concomitant pleural effusion predominantly distributed in the inferior fissure (Fig. 1b)*.* A dynamic fluoroscopic evaluation confirmed the right diaphragmatic paralysis, not present before the procedure We changed the intravenous antibiotic therapy to piperacillin/tazobactam and tigecycline, administered corticosteroid i.v. and high flow oxygen by nasal cannula. After an initial phase of substantial instability, after 4 days the clinical condition and radiological imaging had improved with restored mobility of the right hemidiaphragm. Six months later, the trend of FEV_1_ had been reduced by 15 points compared with FEV_1_ pre-BAE and the patient was included on a waiting list for lung transplantation. Nine months later, she required mechanical ventilation, and subsequently the initiation of extracorporeal membrane oxygenation (ECMO) for a pulmonary exacerbation with septic shock. Lung transplantation in ECMO was performed with success.

## Discussion and conclusions

We report the first case of a pediatric CF patient with massive hemoptysis presenting a phrenic nerve injury following BAE. Similarly, Chapman et al. described a case of unilateral diaphragmatic paralysis following BAE in a 29 year-old woman due to a non-target embolization as the inadvertent obstruction of the pericardiacophrenic artery.^4^ In our case the angiography did not show any connection of the right bronchial artery to other vessels (mammary artery, phrenic artery) or any connection to the circle of the phrenic nerve (Fig. 1a). However, we recommend the importance of considering acute pulmonary embolism in the patient’s differential diagnosis.

Diaphragm dysfunction is typically due to phrenic nerve dysfunction, which can have various origins: malignancy infiltration, nerve lesion during thoracic surgery and diseases affecting peripheral or central nervous systems.^5^ In many cases, isolated phrenic neuropathy with no apparent cause is categorized as idiopathic. Infectious processes have been proposed as the underlying cause.^5^

Diaphragm dysfunction may be underdiagnosed, but it can negatively affect quality of life, can be a marker of disease severity and, in some instances, such as in the intensive care unit, be a prognostic marker.

BAE is undoubtedly a lifesaver procedure in massive hemoptysis, but it can lead to severe complications also in pediatric age. The risk/benefit ratio must be carefully evaluated. In particular, the end-stage CF can represent an important risk factor. Clinicians should be aware of the possibility of phrenic nerve injury with BAE monitoring the appearance of chest pain and dyspnea after the procedure since this complication is unpredictable and no special cares are known to avoid it.

## Data Availability

The datasets used and/or analyzed during the current study are available from the corresponding author on reasonable request.

## Abbreviations

BAE: Percutaneous bronchial artery embolization
CF: Cystic fibrosis
CTA: Computed tomographic angiography
ECMO: extracorporeal membrane oxygenation
FEV_1_: Forced expiratory volume in 1 s

## References

[1] Flume PA, Yankaskas JR, Ebeling M, Hulsey T, Clark LL (2005). Massive hemoptysis in cystic fibrosis. Chest.

[2] Flume PA, Mogayzel PJ, Robinson KA, Rosenblatt RL, Quittell L, Marshall BC (2010). Cystic fibrosis pulmonary guidelines: pulmonary complications: hemoptysis and pneumothorax. Am J RespirCrit Care Med.

[3] Cody O'Dell M, Gill AE, Hawkins CM (2017). Bronchial artery embolization for the treatment of acute hemoptysis. Tech Vasc Interv Radiol.

[4] Chapman SA, Holmes MD, Taylor DJ (2000). Unilateral diaphragmatic paralysis following bronchial artery embolization for hemoptysis. Chest..

[5] Dubé Bruno-Pierre, Dres Martin (2016). Diaphragm Dysfunction: Diagnostic Approaches and Management Strategies. Journal of Clinical Medicine.

